# A Metabolomic Approach to Assess the Toxicity of the Olive Tree Endophyte *Bacillus* sp. PTA13 Lipopeptides to the Aquatic Macrophyte *Lemna minor* L.

**DOI:** 10.3390/toxics10090494

**Published:** 2022-08-25

**Authors:** Evgenia-Anna Papadopoulou, Katerina Giaki, Apostolis Angelis, Alexios-Leandros Skaltsounis, Konstantinos A. Aliferis

**Affiliations:** 1 Laboratory of Pesticide Science, Department of Crop Science, Agricultural University of Athens, 11855 Athens, Greece; 2 Department of Pharmacognosy and Natural Products Chemistry, Faculty of Pharmacy, University of Athens, 15771 Athens, Greece; 3 Department of Plant Science, Macdonald Campus, McGill University, Montreal, QC H9X 3V9, Canada

**Keywords:** ecotoxicology, ecotoxicogenomics, endophytic microorganisms, fengycins, iturins, natural products, plant protection, surfactins

## Abstract

Pesticides represent a major human input into the ecosystem, posing a serious risk to non-target organisms. Therefore, there is pressure toward the reduction in their use and the discovery of alternative sources of bioactivity. Endophytic microorganisms represent a source of bioactivity, whose potential for plant protection has been recently established. In this context, an olive tree endophytic *Bacillus* sp. was isolated, exhibiting superior antifungal activity, mainly attributed to its major surfactin, iturin, and fengycin and the minor gageotetrin and bacilotetrin groups of lipopeptides (LP). Based on the potential of LP and the lack of information on their toxicity to aquatic organisms, we have investigated the toxicity of an LP extract to the model macrophyte *Lemna minor* L. The extract exhibited low phytotoxicity (EC_50_ = 419 μg·mL^−1^), and for the investigation of its effect on the plant, GC/EI/MS metabolomics was applied following exposure to sub-lethal doses (EC_25_ and EC_50_). Results revealed a general disturbance of plants’ biosynthetic capacity in response to LP treatments, with substantial effect on the amino acid pool and the defense mechanism regulated by jasmonate. There are no previous reports on the phytotoxicity of LP to *L. minor*, with evidence supporting their improved toxicological profile and potential in plant protection.

## 1. Introduction

Conventional plant protection products (PPPs) still represent a main pilar of the agri-food sector and an essential component in the collective effort to secure food supply and quality for the fast-growing human population [1,2]. However, the major issues that the agrochemical sector is facing, such as climate change, strict regulations related to pesticide registration, the development of resistant pest and pathogen populations, as well as the detection of PPP residues in the food and environment, necessitate the adaptation of new, alternative plant protection strategies based on results of cutting-edge research [3,4,5]. Focusing on the residues, the continuous release of vast amounts of PPPs into the agroecosystems results in the exposure of non-target organisms to various xenobiotics, with the final short and long-term impacts being difficult to be assessed. Many active ingredients (a.i.) of PPPs end up in the surface and/or underground waters, with the most toxic ones substantially affecting the aquatic organisms [6,7,8]. Therefore, the assessment of the risk of a.i. to such organisms in the early stages of their R&D is of paramount importance for the development of novel/alternative PPPs with improved toxicological profiles.

Based on the aforementioned, the discovery of new sources of bioactivity for applications in plant protection that exhibit improved efficacy, low toxicity to human and non-target organisms, and ideally new modes-of-action (MoA), represents the ultimate goal of the agrochemical sector. In the last decade, endophytes have emerged as a promising group of microorganisms of great potential for applications in the agri-food sector, including, among others, plant nutrition and plant protection [9,10,11]. They are described as microbes that, at least for some part of their life cycle, internally colonize plant tissues, inter- or intracellularly, without causing any visible disease symptoms [12,13]. They develop a complicated interaction with their hosts and benefit them to a great extent by promoting their growth, enhancing their resistance to abiotic stresses, and eliciting their defence responses against pathogen infections [10,14,15]. Furthermore, among their main features is the ability to synthesize a vast array of metabolites with original structures that exhibit unique bioactivities [16,17,18].

In this context, the overarching goal of our research is the exploitation of endophytes either *per se* as biological control agents or as sources of bioactive metabolites due to their superior metabolite-producing capacity. In a recent investigation, we obtained a lipopeptide (LP) extract, produced by an olive tree endophytic *Bacillus* sp. isolate, which exhibits antimicrobial properties, and thus its efficacy was assessed against olive tree pathogens [19]. The extract contains various major LP groups, such as surfactins, bacillomycins, and fengycins, as well as the minor gageotetrins and bacilotetrins. Although these LPs exhibit well-studied antimicrobial activities [19,20,21,22], information on their phytotoxicity is limited and largely fragmented, suggesting low phytotoxicity for the ones that have been assessed [23,24]. Nonetheless, corresponding information on their effects on the metabolism is non-existent. Based on the properties and the potential of LPs as plant protection agents, we have undergone the task to investigate their toxicity to non-target organisms, focusing on aquatic ecosystems.

The study of the toxicity of a bioactive agent under investigation to non-target organisms represents a bottleneck towards its further development as PPP. More specifically, according to the Regulation (EC) No 1107/2009 of the European Parliament and of the Council [https://eur-lex.europa.eu/legal-content/EN/TXT/?uri=celex%3A32009R1107 (accessed on 7 August 2022)], ecotoxicological studies for the risk assessment of such an agent to non-target organisms, such as birds, mammals, aquatic organisms, arthropods, and other terrestrial organisms, are prerequisite. For the above-mentioned studies, model organisms, whose responses to the exposure to a bioactive compound can provide insights into its potential effect on higher organisms, are employed.

Here, *Lemna minor* L., which is an aquatic macrophyte and one of the most important model species in ecotoxicology due to its favorable traits, was used for the assessment of the LP extract’s phytotoxicity performing GC/EI/MS metabolomics. Although the toxicity of a few LPs has been studied to aquatic organisms, such as *Daphnia* sp. and *Artemia* sp. [25], to the best of our knowledge, there are no previous studies on their effects on *L. minor* L. applying metabolomics for the dissection of the cascade of the undergoing events at the metabolite level associated with the corresponding toxicity. The minute size and simple morphology of the plant enable its cost-effective cultivation and handling under limited laboratory conditions, while the fast production of uniform populations represents a significant advantage for the application of omics approaches. The latter has been confirmed in high-throughput metabolomics studies, where the plant’s metabolism fluctuated in response to treatments with a wide range of xenobiotics and several metabolites–biomarkers of effect being discovered [26,27]. In these studies, an excellent correlation was documented between the toxicity of xenobiotics being tested and parameters, such as the photosynthetic pigment content, number of fronds, and produced biomass. The Organization for Economic Co-operation and Development (OECD) and the International Organization for Standardization (ISO) have developed protocols and guidelines for the use of the plants as a reference organism in toxicity risk assessment [28,29]. Furthermore, EFSA recommends *L. minor*, as well as other species of the genus *Lemna*, in risk assessment studies of PPPs.

## 2. Materials and Methods

### 2.1. Chemicals and Reagents

The LP extract being assessed had been obtained by liquid cultures of the olive tree endophytic *Bacillus* sp. PTA13 in Lysogeny Broth medium, as previously described [19], and it is a mixture of major (e.g., surfactin, bacillomycin, fengycin) and minor (e.g., gageotetrin, bacilotetrin) LP groups. A stock solution of the extract (50,000 ppm) was initially prepared in dimethyl sulfoxide (DMSO, Chem-Lab NV, Zedelgem, Belgium) into 2 mL Eppendorf tubes and it was kept at −30 °C. DMSO was also used in the extraction of photosynthetic pigments of the plant. Ethyl acetate (EtOAc) and methanol (MeOH) (GC/MS grade, 99.9% purity, Carlo Erba Reagents, val de Reuil, France) were used for the extraction of *Lemna*’s metabolome. Reagents that were used in sample preparation for GC/EI/MS metabolomics, such as pyridine (99.8%, *v*/*v*), methoxylamine hydrochloride (98% *w*/*w*) and ribitol, were purchased from Sigma-Aldrich Ltd. (Steinheim, Germany), and N-methyl-N-(trimethyl-silyl) trifluoroacetamide (MSTFA) from Macherey-Nagel (Düren, Germany).

### 2.2. Cultivation of Lemna minor L.

Axenic *L. minor* cultures were grown in 100 mL of Steinberg medium (SM) into 1 L glass bottles following standard operating procedures (SOPs) according to the protocol 221 of the Organization for Economic Co-operation and Development (OECD) [28]. The composition of the medium is displayed in the Tables S1 and S2. For the preparation of the stock solutions, the solutions listed in the Tables S1 and S2 were prepared in sterile glass bottles using sterile deionized water. Stocks #1–7 were autoclaved, whereas stock #8 was filter-sterilized (0.2 μm in diameter). The stock solutions were kept at 4 °C until further use. The pH of the medium was adjusted to 5.5 ± 0.2. All handling was performed under aseptic conditions in a Laminar flow cabinet hood.

Regular subculturing was performed biweekly by transferring three to four healthy plants to new culture medium. The plants were then incubated in a growth chamber at 22 ± 1 °C, under continuous light at an approximate intensity of 110 μmol·m^−2^·s^−1^, and relative humidity (RH) of 85 ± 5%.

### 2.3. Bioassays for the Assessment of the EC_25_ and EC_50_ Values of the Bacillus sp. PTA13 Lipopeptide (LP) Extract to Lemna minor L.

For the mining of the phytotoxicity of LP extract to *L. minor*, an initial screening is necessary in order to determine the EC_25_ and EC_50_ values. For the bioassays, two healthy and uniform *L. minor* plants were transferred into the wells of a 12-well sterile plastic culture plate containing LP solutions at a range of concentrations. The solutions were prepared by adding an appropriate quantity of LP stock solution in SM. Six different LP concentrations were tested in triplicates (1, 10, 50, 100, 250, and 500 ppm). In order to test the effect of DMSO on the growth of the plant, plants were also cultured in SM supplemented with 1% *v*/*v* DMSO. Plants were incubated for 72 h as described above (§2.2). Then, they were harvested, and the impact of LP was assessed based on the measurement of four parameters: fresh weight, number of fronds, and chlorophyll, and carotenoid contents.

The fresh weight of the plants was recorded following the removal of residual SM by absorbent paper. Then, they were immediately placed into Eppendorf tubes (2 mL) and their metabolism was quenched by dipping into liquid N. The material was stored at –80 °C until further analysis. For the extraction of photosynthetic pigments, although various protocols have been developed, using DMSO was used, since it is simple and fast without requiring grinding and further processing of the samples [30]. Briefly, samples were extracted into 1 mL of DMSO in 2 mL Eppendorf tubes in a water bath at 65 °C for 30 min. Then, the extracts were removed and their optical density was recorded for various wavelengths (480, 645, 663, and 665 nm) using a Uvikon 922 spectrophotometer (Kontron Instruments, Augsburg, Germany). Based on the measurements, the content (mg·mL^−1^) of the tissues in chlorophylls *α* and *β*, total chlorophyll and carotenoids were calculated [31,32].

Statistical analysis was performed applying the Student’s *t*-test (*p* > 95%) using the software JMP Pro v.16.1.0 (SAS Institute, Cary, NC, USA). The EC_50_ values were calculated according to the Probit method.

### 2.4. Mining the Effect of the Bacillus sp. PTA13 Lipopeptide Extract on the Metabolism of Lemna minor L. by GC/EI/MS Metabolomics

#### 2.4.1. Experimental Design

*Lemna* plants were grown in SM containing LPs at the concentration of EC_25_ or EC_50_, based on the results of bioassays (§2.3). These concentrations for the LP were selected because a metabolomics investigation requires the exposure of the biological system under study to sub-lethal doses of a bioactive agent in order to facilitate a minimum disturbance of its metabolism. Additionally, plants were grown in SM containing 1% *v*/*v* DMSO. For each treatment, 15 biological replications of 2 plants each were performed in sterile 12-well cell culture plates. All handling was performed aseptically in a laminar flow cabinet following SOPs. The plants were placed in a growth chamber and were incubated as described in §2.2 for 72 h. Then, they were removed from the chamber and macroscopic observations were taken. Next, their preparation for metabolomics analysis followed as described below.

#### 2.4.2. Sample Preparation for GC/EI/MS Metabolomics Analysis

Every three biological replications were grouped to obtain one pooled sample. Plants were rinsed in deionized water to remove possible residues and dried using absorbent paper. The weight of the pooled samples was quickly recorded, and they were put into 2 mL Eppendorf tubes, which were immediately immersed into liquid N for metabolism quenching. Samples were pulverized to a fine powder in a mortar under liquid N and the metabolome of the plant was extracted following a previously described protocol, with minor modifications, using 1 mL of a MeOH:EtOAc mixture (1:1 *v*/*v*). To improve the extraction performance, the samples were sonicated for 20 min in an ultrasonic bath (Branson 1210, Danbury, CT, USA) and then stirred in a horizontal orbital shaker (GFL 3006, Geschacha für Labortechnik mbH, Burgwedel, Germany) for 1 h at 150 rpm, at room temperature. For the removal of particles, extracts were filtered using 0.2 μm in pore-diameter filters (Macherey-Nagel, Duren, German) and 20 μL of a ribitol solution (0.2 mg mL^−1^ in methanol) were added as internal standard. Evaporation of the solvent followed using a refrigerated vacuum concentrator (Labconco, Kansas City, MO, USA) equipped with a cold trap.

The dried extracts were derivatized in a two-step process, following a previously described protocol [26]. Briefly, 80 μL of a methoxylamine hydrochloride solution 20 mg mL^−1^ in pyridine was added, followed by incubation in a water bath at 30 °C for 2 h (Daihan Labtech, Gyeonggi-do, Korea) under continuous agitation. This step aims at the stabilization of sugars in order to obtain improved chromatograms [33]. Then, 80 μL of MSTFA were added and the extracts were incubated at 37 °C for 90 min in a water bath under continuous agitation. During silylation, trimethylsilyl (TMS) moieties replace active hydrogens of functional groups of metabolites, thus, substantially improving their volatility and stability [33]. The derivatized extracts were finally transferred into 200 μL glass micro inserters (Macherey-Nagel) in glass autosampler vials (2 mL) for GC/EI/MS analysis. In order to discover features not related to the analyzed plant material, experimental blanks were prepared following identical handling to that of the biological pooled samples.

#### 2.4.3. GC/EI/MS Analytical Conditions

An Agilent 6890 MS (Agilent Technologies Inc., Santa Clara, CA, USA) platform equipped with a 5973 inert mass selective detector (MSD) was employed for the recording of the plants’ metabolomes. The derivatized extracts (1 μL) were injected on column (HP-5MS, 30 m long, 0.25 mm diameter, 0.25 μm film thickness, Agilent Technologies Inc.) at a split ratio 5:1. The temperature of the injector was set at 230 °C and helium (He) was used as the carrier gas at a flow rate of 1 mL·min^−1^. The initial temperature of the oven was set at 70 °C, then it remained stable for 5 min following a 5 °C·min^−1^ rate increase until 310 °C and was finally kept stable for 1 min. Positive electron ionization was used (70 eV) and full scan mass spectra were acquired in the mass range 50–800 Da (4 scans sec^−1^), with an initial signal recording delay of 6 min. The temperature of the MS source was set at 230 °C and that of the quadrupole at 150 °C. All experimental events were controlled using the Agilent’s MSD ChemStation E.02.01.1177 software (Agilent Technologies Inc., Santa Clara, CA, USA).

#### 2.4.4. Data Pre-Processing and Biomarker Discovery

The obtained total ion chromatograms (TIC) were initially deconvoluted using the software AMDIS v.2.66 and the mass spectra library of NIST 08 (National Institute of Standards and Technology library, NIST, Gaithersburg, MD, USA). Tentative identification of detected metabolite features was performed based on the matching of their mass spectra to those of known compounds with similarity > 95%. For selected metabolites, absolute identification was performed using analytical standards that were analyzed in the same system under identical conditions [34].

For the detection of trends within the treatments and the discovery of the corresponding biomarkers of LP toxicity to *Lemna*, a previously described bioinformatics pipeline was followed [26]. Initially, the software MS DIAL v.4.90 [35] was employed for the TIC preprocessing (e.g., baseline correction, alignment, feature removal). The obtained matrix was exported to Microsoft Excel^®^ for further curation and addition of information for the annotated metabolites (e.g., coding, biosynthetic pathways). The curated matrix was then exported to Simca SIMCA-P+ software v.16.0. (Umetrics, Sartorius Stedim Data Analytics AB, Umeå, Sweden) for multivariate analyses as previously described [26], in order to discover the metabolites–biomarkers of LP toxicity. Additionally, the matrix was visualized and analyzed creating heatmaps, using the software Matlab v.R2022a (The MathWorks, Inc., Natick, MA, USA).

## 3. Results and Discussion

### 3.1. The Chlorophyl α Content Is the Most Sensitive Indicator of the Bacillus sp. Lipopeptide Extract’s Toxicity to Lemna minor L.

**Figure 1:**
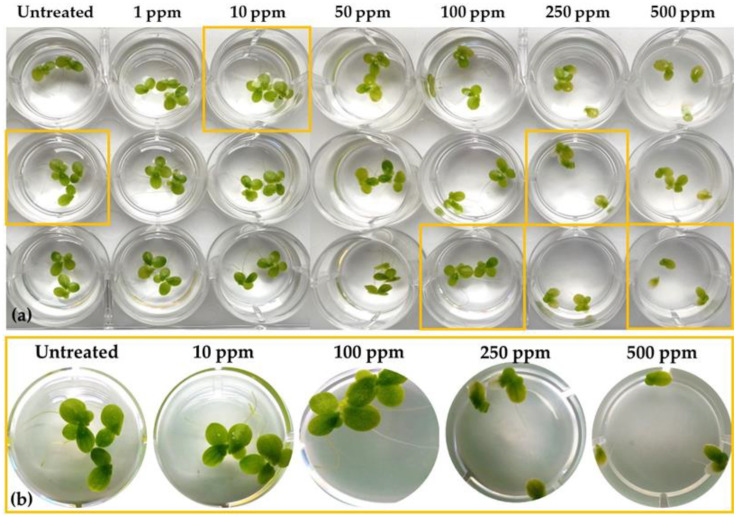
(**a**) Phenotypic observations of *Lemna minor* L. cultures 72 h post-treatment with the *Bacillus* sp. PTA13 lipopeptide extract. The extract was applied at the concentrations of 1, 10, 50, 100, 250, and 500 μg·mL^−1^, performing three biological replications per treatment. (**b**) Magnification of representative treatments enables the visualization of the effects of the lipopeptide extract on the plant.

**Figure 2:**
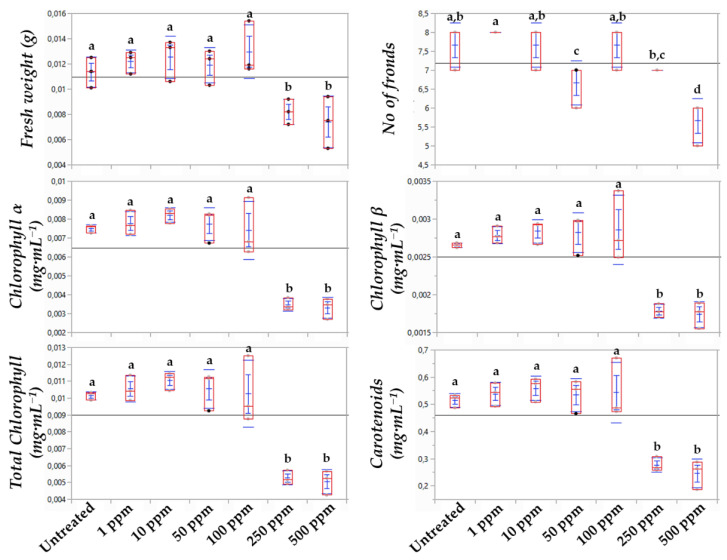
Box plots demonstrating the effect of the *Bacillus* sp. PTA13 lipopeptide extract on the fresh weight, number of fronds, and chlorophyll (*α*, *β* and total) and carotenoid contents of *Lemna minor* L. 72 h following treatment. The extract was applied at 1, 10, 50, 100, 250, and 500 μg·mL^–1^ and three biological replications were performed per treatment. The different letters above boxes indicate statical significance (Tukey HSD test, *p* > 95%).

For the assessment of the toxicity of LPs to *Lemna* applying metabolomics, bioassays for the estimation of the EC_25_ and EC_50_ values were initially performed. The effect of a range of LP concentrations on the phenotypes and number of fronds, the fresh weight, and the content of plants in chlorophylls and carotenoids was recorded (Figure 1 and Figure 2). Based on the statistical analyses and the observed phenotypes, the content of plants in chlorophyll *α* proved to be the most sensitive indicator of the LP toxicity, with EC_25_ and EC_50_ values of 156 ppm and 419 ppm, respectively. The corresponding values based on the LPs’ effect on the biomass were 528 and >500 ppm, respectively, and based on the effect on chlorophyll *β*, 272 ppm (EC_25_) and >500 ppm (EC_50_). The EC_25_ based on the effect on total chlorophyll and carotenoids were 188 and 74, respectively, and the corresponding EC_50_ values were 521 and 657 ppm. Plants’ chlorophyll and carotenoid contents are known as sensitive indicators of their photosynthetic activity, developmental changes, as well as responses to stresses [36]. Although in *Lemna* bioassays, parameters related to the development of the plants, such as number of fronds and the production of biomass, are commonly used, bioassays based on the assessment of the effect on chlorophyll content are highly sensitive for the testing of organic aquatic micropollutants. The latter is in agreement with our finding that the chlorophyl *α* content is the most sensitive indicator of the *Bacillus* sp. LP extract’s toxicity to *L. minor* [37].

### 3.2. Overview of the Metabolomics Analysis

**Figure 3:**
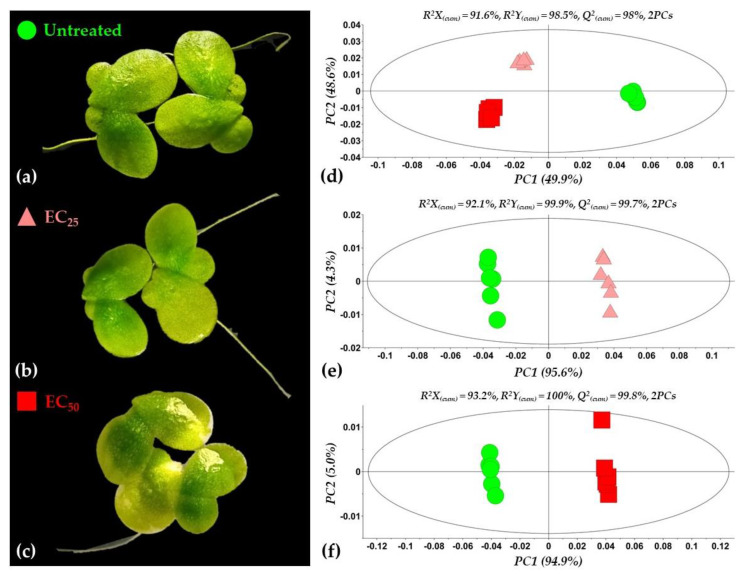
Effect of the *Bacillus* sp. PTA13 lipopeptide extract on the phenotypes of *Lemna minor* L. (**a**–**c**) and the corresponding GC/EI/MS metabolite profiles (**d**–**f**). The plants were exposed to concentrations equal to the EC_25_ (156 μg·mL^−1^) and EC_50_ (419 μg·mL^−1^) of the extract and analyses were performed 72 h following exposure. Both treatments resulted in a slight discoloration of the plants’ fronds (**b**,**c**). The metabolite differences between the untreated and the lipopeptide-treated plants were analyzed applying orthogonal partial least squares discriminant analysis (OPLS-DA). OPLS-DA PC1/PC2 score plots are displayed with 95% confidence interval. The ellipse represents the Hotelling’s T^2^. In total, fifteen biological replications of two plants each were performed per treatment, every three of which were pooled to provide a pooled sample. Five pooled and one quality control samples were analyzed per treatment [PC; principal component, Q^2^_(cum)_; cumulative fraction of the total *X*’s variation that can be predicted, R^2^X and R^2^Y; fraction of the sum of squares of *X*’s and *Y*’s explained by the components, respectively].

**Figure 4:**
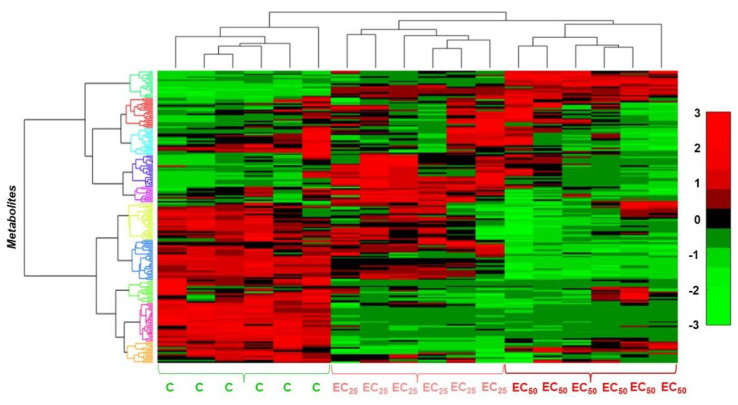
Effect of the *Bacillus* sp. PTA13 lipopeptide extract (EC_25_ = 156 μg·mL^−1^ and EC_50_ = 419 μg·mL^−1^) on the GC/EI/MS metabolite profiles of *Lemna minor* L. 72 h following treatments, visualized using two-dimensional cluster heat map. Hierarchical cluster analysis (HCA) was performed applying the Ward’s linkage method. The columns correspond to the treatments and the rows to metabolite features. Cells are color-coded according to the relative content of the metabolite features ranging from −3 (light green) to 3 (light red), designating low to high values, respectively. In total, fifteen biological replications of two plants each were performed per treatment, every three of which were pooled to provide a pooled sample. Five pooled samples and one quality control were analyzed per treatment (C; untreated).

The applied bioanalytical protocol resulted in an improved recording of *Lemna*’s GC/EI/MS metabolite profiles following treatments of the plant with the LP extract (Figure S1) as revealed by the obtained total ion chromatograms (Figure S2); an excellent chromatographic separation was observed among the various recorded metabolite features, the obtained peaks were sharp and symmetric, and an optimum baseline was observed throughout the analysis. A representative data set can be accessed via the website of the Pesticide Metabolomics Group at https://www.aua.gr/pesticide-metabolomicsgroup/Resources/default.html (*Lemna minor* L., PMG-01-2022). The robustness of the applied protocols was further confirmed by the tight grouping of the biological replications of each treatment performing multivariate (Figure 3) and heatmap analyses (Figure 4).

**Figure 5:**
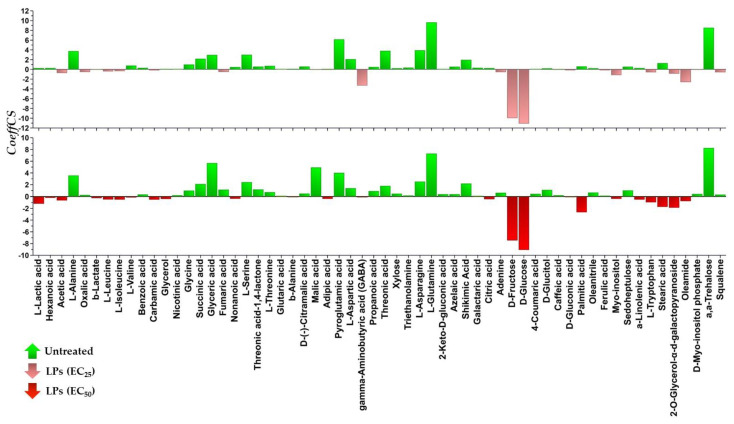
Coefficient plots displaying metabolite differences between the untreated and EC_25_ (156 μg·mL^−1^) (**Upper plot**) or EC_50_ (419 μg·mL^−1^) lipopeptide-treated (**Lower plot**) *Lemna minor* L. plants. Values of scaled and centered OPLS regression coefficients (CoeffCS) are displayed. High absolute values of CoeffCS correspond to annotated metabolites with the highest leverage on the observed discrimination. Positive values correspond to metabolites with increased levels in the untreated, whereas negative to those with increased levels in the lipopeptide-treated plants.

**Figure 6:**
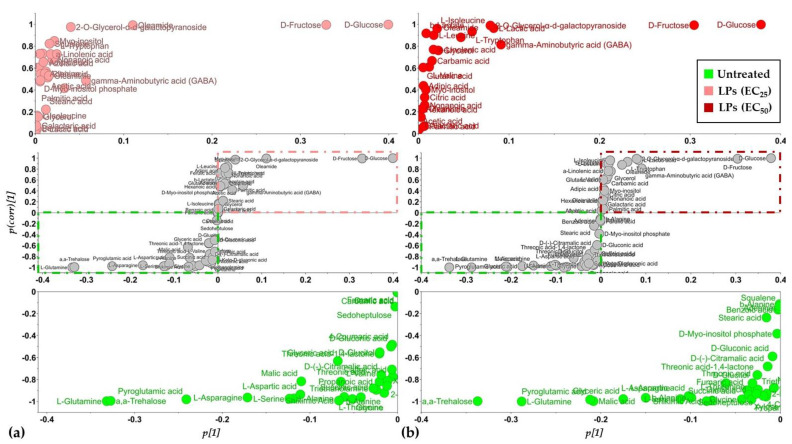
S-plots for the visualization of the OPLS-DA predictive component loadings for the study of the toxicity of the *Bacillus* sp. PTA13 lipopeptide extract on the metabolism of *Lemna minor* L. The plants were exposed to concentrations equal to the EC_25_ (156 μg·mL^−1^) (**a**) and EC_50_ (419 μg·mL^−1^) (**b**) of the extract and analyses were performed 72 h following exposure. Metabolites located far out on the wings of the S-plot correspond to biomarkers of lipopeptide toxicity. The S-plots are displayed in the middle panels and magnification of the upper right (upregulated metabolites) and lower left (downregulated metabolites) quadrants are displayed in the upper and lower panels, respectively.

**Figure 7:**
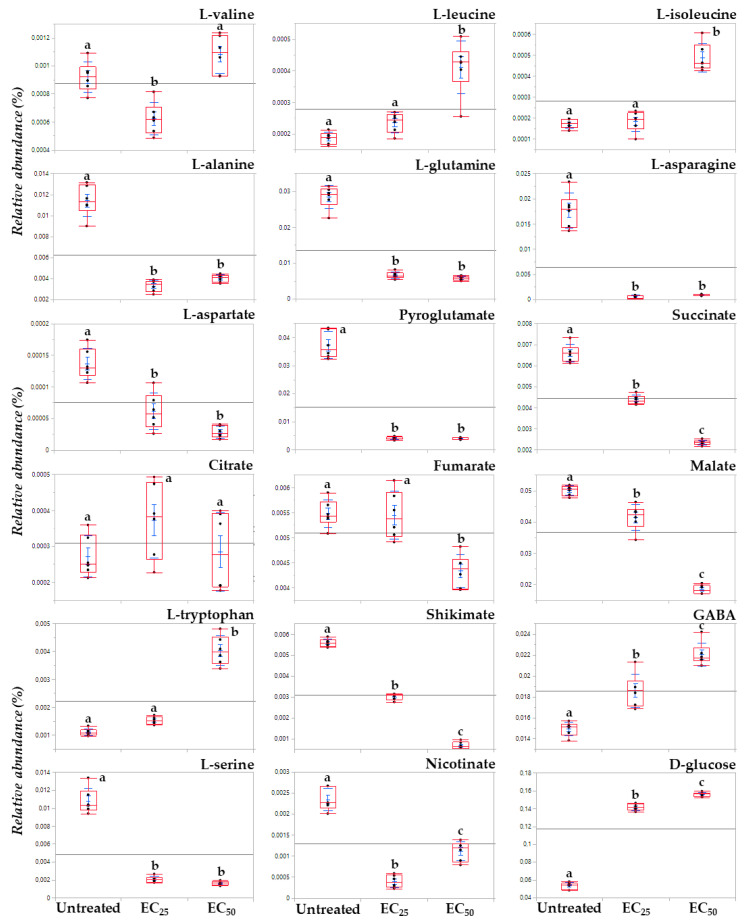
Box plots demonstrating the effect of the *Bacillus* sp. PTA13 lipopeptide extract on selected annotated *Lemna minor* L. metabolites 72 h following treatment with concentrations equal to the EC_25_ (156 μg·mL^−1^) and EC_50_ (419 μg·mL^−1^) values. In total, fifteen biological replications of two plants each were performed per treatment, every three of which were pooled to provide a pooled sample. Five pooled samples and one quality control sample were analyzed per treatment. The different letters above boxes indicate statical significance (Tukey HSD test, *p* > 95%).

Following the removal of metabolite features that were present in less than the 75% of the recorded profiles, a matrix composed of 204 rows was obtained. In total, 86 metabolite features were annotated in various identification levels (Table S3). Both treatments of plants with concentrations equal to the EC_25_ and the EC_50_ of the extract had a substantial effect on their metabolism, resulting in distinct metabolite profiles (Figure 3d–f and Figure 4). Various metabolites that belong to different chemical groups and contribute to the observed clustering were discovered based on the values of scaled and centered OPLS regression coefficients (CoeffCS), S-plot analysis, and ANOVA (Figure 5, Figure 6 and Figure 7) and the values of the variable influence on projection (VIP) plots (Figures S3 and S4). Overall, there was a correlation observed between the effects of LP extracts on the phenotypes and the metabolism of the plant. Although a large number of metabolites–biomarkers was discovered, only those with the highest fluctuation between untreated and LP-treated plants that additionally play key roles in plant’s metabolism will be discussed next.

### 3.3. The Lipopeptide Extract of the Olive Tree Endophytic Bacillus sp. PTA13 Substantially Affects the Amino Acid Pool, the Energy Equilibrium and the Levels of Metabolites That Play Key Roles in the Physiology of Lemna minor L. Plants

Treatment of *Lemna* with the LP extract at concentrations equal to its EC_25_ (156 μg·mL^−1^) or EC_50_ (419 μg·mL^−1^) values resulted in distinct phenotypes, with untreated plants clearly exhibiting higher content in photosynthetic pigments compared to the LP-treated ones (Figure 3a–c). Following 72 h of exposure to the EC_25_, plants developed slight chlorosis and occasionally inhibition of new fronds’ development was observed. On the other hand, following treatment with the high LP concentration (EC_50_), the symptoms of toxicity were moderate, with plants developing apical discoloration. These distinct phenotypes were confirmed by the corresponding metabolite profiles of the plants as discussed below.

#### 3.3.1. Fluctuation of the Amino Acid (AA) Pool of *Lemna minor* L.

Treatment of the plant with the LP extract substantially affected its amino acid (AA) pool (Figure 5, Figure 6, Figure 7, Figures S3 and S4), which agrees with references on their role as indicators of plants’ physiological condition [38] and results from previous works on the effect of xenobiotics on *Lemna* [26,39,40]. AA are the building blocks for the biosynthesis of metabolites and proteins, and, additionally, they play multiple roles in plant metabolism, being components of the signaling and stress response mechanisms [41,42].

Interestingly, the content of *Lemna* plants for the vast majority of the annotated AA decreased in response to LP exposure. Among these, the AA of the glutamate family; glutamine and pyroglutamate of the aspartate family aspartic acid; L-asparagine; L-threonine; and L-serine (Figure 5, Figure 6, Figure 7, Figures S3 and S4).

Such decreased levels in the majority of AA possibly indicate low availability of these building blocks for the biosynthetic needs of the plant, which seems to be the result of the reduced activity of *Lemna’s* photosynthetic apparatus and the related processes. In response to the LP toxicity, the plant seems to suppress its biosynthetic activity in order to preserve energy and at the same time it favors the operation of catabolic pathways in order to generate energy; these are results that are in line with previous reports [43]. Such hypothesis is further supported by the increased levels of glucose in the LP-treated plants that possibly indicates the reduced operation of the glycolytic pathway, by which the carbohydrate is oxidized to pyruvate and that of the Krebs cycle as discussed below (§3.3.3.). The AA of the glutamate family marked a substantial decrease following plants’ treatment with the LP extract. These AA play important role in the N and energy metabolism, and biosyntheses [44]. Moreover, glutamine is a precursor for chlorophyll and heme [45]. The fluctuations in the levels of these metabolites following the LP treatments confirm the important roles in *Lemna*’s metabolism being among the ones with the highest leverage to the observed discrimination with the untreated plants. AA of the aspartate family have a cornerstone role in plants’ metabolism mainly being associated with the energy production via the Krebs cycle [46]. Representatives of this family, such as aspartate participates in various biosynthetic pathways, being among the key metabolites in plants’ metabolism [47]. The decreased levels of the metabolites in the LP-treated plants further confirm their deleterious effect on *Lemna*. L-serine is another AA that plays a key role in plants and is synthesized via various biosynthetic pathways with the photorespiratory glycolate pathway being the primary [48]. This AA is essential element of various biosyntheses, cell proliferation, signaling, and responses to stresses [26,48,49]. The dramatic decrease in L-serine following treatments with the LPs possibly indicates its reduced biosynthesis via the photorespiratory glycolate pathway, and it is in agreement with a previous study on herbicides, which also caused reduced levels of L-serine in *Lemna* plants. Nonetheless, increased levels of L-serine in plants have been recorded in response to abiotic stresses [50].

On the other hand, a handful of the annotated AA were increased following treatment with the LP extract, such as those of the pyruvate family L-valine, L-leucine, and L-isoleucine, except for L-alanine, the aromatic AA L-tryptophan, and the non-protein amino acid GABA.

L-tryptophan is an aromatic AA synthesized via the shikimate biosynthetic pathway, which is fueled by glycolysis and the pentose phosphate pathway, and it is a key component in the biosynthesis of proteins and numerous secondary plant metabolites [51], such as alkaloids, phenylpropanoids, glucosinolates, and plant hormones. Therefore, it plays a cornerstone role in plants’ metabolism regulation, physiology, and responses to various stimuli. A substantial increase in the levels of the metabolite (3.7-fold) was recorded in plants treated with the EC_50_ concentration of the LP, whereas a moderate increase (1.4-fold) is found following their exposure to the EC_25_ concentration (Figure 5, Figure 6, Figure 7, Figures S3 and S4). Such fluctuation was accompanied by a substantial decrease in its precursor, shikimate, whose relative concentration marked a 1.8- and 8.4-fold decrease following treatments with the EC_25_ and EC_50_, respectively. This observation plausibly indicates that LPs at the concentration of 419 ppm cause a substantial downregulation of the secondary metabolism of the plant that has L-tryptophan as the key precursor metabolite (e.g., alkaloids, phenylpropanoids, plant hormones), and seems to be a mechanism by which LPs exert their toxicity, causing a diversion of the plants’ biosynthetic mechanism. Increased levels in *Lemna* have been also observed following treatments of the plant with herbicidal active ingredients, including glyphosate that inhibits the biosynthesis of aromatic AA [26]. Nonetheless, the biosynthesis of aromatic AA is under the control of a transcriptional and posttranscriptional regulatory mechanism, whose operation is largely unknown.

Furthermore, the content of the plants in the ubiquitous non-protein amino acid GABA increased following treatments with the LP extract at the EC_25_ and EC_50_ concentrations (Figure 5, Figure 6 and Figure 7). Interestingly, such an increase was higher following treatments with the former. GABA plays a central role in plant metabolism, such as the regulation of C:N balance, responses to biotic and abiotic stresses, stomatal function, or pH regulation [42,52,53]. Nonetheless, although the metabolite had been initially viewed in the context of plant responses to stresses, recent evidence suggests its role as a key signal plant metabolite [52]. In plants, it is metabolized via the GABA shunt, which bypasses the tricarboxylic acid (TCA) cycle and plays multiple roles in their metabolism, including responses to stresses [42,52]. Although the metabolism of GABA is complex, the fluctuations in its content caused by the different LP concentrations being applied, possibly indicates the disturbance of the function of the GABA shunt and/or the polyamine biosynthetic pathway, or the degradation of the molecule for the protection against oxidative stress. The latter has been reported in the plant pathogenic fungi *Magnaporthe grisea* and *Phytophthora infestans* following treatment with the cyclic lipopeptides iturin A and fengycin, which are also contained in the LP extract [53,54]. These LPs caused the onset of reactive oxygen species (ROS) and downregulated ROS-scavenging enzymes.

#### 3.3.2. Induction of Systemic Jasmonate-Mediated Signaling Mechanism of *Lemna minor* L.

Metabolomics analysis revealed an elevated content of the LP-treated plants in *α*-linolenic acid (Figure 5, Figure 6 and Figure 7), which is a key metabolite in plants’ metabolism, being the precursor of jasmonate via the octadecanoid biosynthetic pathway [55,56]. The highest increase was recorded following the treatment of the plants with the LP extract at the EC_50_ concentration (2.6-fold), whereas their treatment with the EC_25_ concentration resulted in a 2.2-fold increase compared to the untreated. The jasmonate-regulated signalling mechanism is one of the two major mechanisms that orchestrate plant responses to biotic and abiotic stimuli [56,57,58,59,60]. Additionally, via the activity of lipoxygenase (LOX), *α*-linolenic acid can be converted into bioactive fatty acid hydroperoxides. The results suggest that the LP extract being applied at both concentrations triggers the systemic jasmonate-mediated signaling mechanism of *Lemna* and agrees with previous studies on plant priming following treatment with cyclic LPs, such as surfactins, iturins, and fengycins [61,62,63,64,65], which are also components of the *Bacillus* sp. PTA13 LP extract. Nonetheless, the cascade of the undergoing molecular events is largely unknown. Although the LP extract exhibits moderate toxicity to *Lemna*, it triggers its defence mechanism, which, from a plant protection perspective, represents a significant advantage towards its further consideration as a potential novel plant protection agent with improved toxicological profile.

#### 3.3.3. Lipopeptides Affect the Energy Equilibrium of *Lemna minor* L.

The disturbance of the plant’s metabolism following treatment with the LP extract was also evident by the fluctuations in its content in the annotated Krebs cycle intermediates (KCI); the levels of fumarate, malate, and succinate marked a substantial decrease following treatments with both the EC_25_ and EC_50_ concentrations, whereas the content in citrate was not affected (Figure 5, Figure 6 and Figure 7), which are indicative of the plant’s energy deprivation with a simultaneous reduced biosynthetic operation. The Krebs cycle is located in the mitochondrial layer and involves a set of reactions where pyruvate is completely oxidized to CO_2_ and bridges the metabolism of carbohydrates and fatty acids with the biosynthesis of components, such as the amino acids and proteins. Additionally, during its operation, energy is being produced in the form of ATP and NADH. The fluctuations in the levels of *Lemna’s* KCI were more pronounced following treatments with the EC_50_. The observation is in agreement with the abovementioned results on the reduced operation of the biosynthetic mechanism of the plant as a consequence of the LP toxicity. KCI have multiple roles, for example malate regulates plant nutrition and growth [66,67], and fumarate is involved in the urea cycle, amino acid metabolism, and the regulation of stomata function [68,69]. Nonetheless, recent evidence suggests an additional role of the KCI as signaling molecules [70] without the obtained evidence being adequate to explain such a role in the current study.

#### 3.3.4. Effect of Lipopeptides in the Content of the Plant in Metabolites with Key Role in Its Physiology

Interestingly, the levels of various annotated metabolites with well-established roles in plants’ responses to stresses, such as nicotinate and the phenolics ferulate, 4-coumarate, and caffeate, were not substantially altered in response to the treatments with the LPs (Figure 5 and Figure 6). Nicotinate is a common form of vitamin B3, involved in photosynthetic processes and plants’ responses to stresses, protection against oxidative stress, and additionally serves as a signaling molecule [71,72,73]. The phenolics caffeate, 4-coumarate, and ferulate, also play multiple roles in plant physiology, such as the responses to various stimuli, and additionally, they exhibit antioxidant, cytotoxic, and antimicrobial properties [74,75,76]. However, based on the acquired evidence, it seems that the mechanisms in which these molecules are involved do not play an essential role in *Lemna*’s responses to the LP-induced toxicity. The results on caffeate are in contrast to those obtained in the response of the plant to selected herbicides and their mixtures [26], plausibly indicating distinct mechanisms of toxicity between these herbicides and the applied LPs.

The non-reducing disaccharide α, α-trehalose, marked a substantial decrease in response to the LP treatments (Figure 5 and Figure 6). It is a ubiquitous metabolite present in high concentrations in a wide range of organisms, including fungi, bacteria, and plants [77,78]. In plants, it is involved in a variety of processes, such as embryo and leaf development [79], flowering, and starch metabolism [80], as well as cell division and cell wall synthesis [81]. In addition, research has demonstrated the role of trehalose in the response to environmental stresses [82] and especially drought conditions [77]. The results possibly suggest an elevated activity of the enzyme trehalase, which catalyzes the breakdown of trehalose to two molecules of glucose, an observation that could partially explain the increased levels of glucose. Similar fluctuation was observed in a previous study following treatment of *Lemna* with individual herbicides and their mixtures [26].

## 4. Conclusions

The agri-food sector is facing a great undertaking in its effort to secure food quality and supply following a sustainable production model. The adaptation of original approaches in plant protection seems to be a key in order to address such challenges, and, in this context, the exploitation of new sources of bioactivity is an alternative to conventional PPPs of high potential. Nonetheless, the assessment of the risk of such bioactivities to non-target organisms is a prerequisite for their further development, and, currently, it represents a bottleneck in their R&D pipeline. Here, the assessment of the phytotoxicity of the *Bacillus* sp. PTA13 LP extract to the model plant *L. minor* L. applying metabolomics, revealed that, although it affects the metabolism of the plant, it is moderately toxic with relatively high values of EC_50_. Such observation is encouraging toward the further development of the product, since the evidence suggests an improved toxicological profile, and, to the best of our knowledge, no similar previous reports exist. However, an obstacle toward the development of the LPs as PPPs seems to be the moderate yield that is usually achieved applying the existing isolation protocols [19,83,84,85]. This can be overcome via the optimization of the culture media/cultivation regime [25,83,86] and/or the employment of genetic engineering approaches [25,87,88,89]. Additionally, further investigation is required in order to mine possible toxic effects of the LPs on other biological systems that could pose a risk to non-target organisms when applied in agroecosystems, as has been the case for several commercial PPPs.

## Data Availability

A representative data set can be accessed via the website of the Pesticide Metabolomics Group at https://www.aua.gr/pesticide-metabolomicsgroup/Resources/default.html (*Lemna minor* L., PMG-01-2022).

## Supplementary Materials

The following supporting information can be downloaded at: https://www.mdpi.com/article/10.3390/toxics10090494/s1, Figure S1: Phenotypic observations of *Lemna minor* L. cultures 72 h post-treatment with the *Bacillus* sp. PTA13 lipopeptide extract or untreated. The extract was applied at concentrations equal to the EC_25_ (156 μg·mL^−1^) and EC_50_ (419 μg·mL^−1^). In total, fifteen biological replications of two plants each were performed per treatment, every three of which were pooled to provide a pooled sample. Five pooled samples and one quality control sample were analyzed per treatment. Figure S2: Representative GC/EI/MS total ion chromatograms (TIC) of *Lemna minor* L. plants; untreated and lipopeptide-treated at concentrations equal to the EC_25_ (156 μg·mL^−1^) and EC_50_ (419 μg·mL^−1^). Figure S3: Variable influence on projection (VIP) plot displaying the VIP values of the annotated *Lemna minor* L. metabolites. High VIP values correspond to metabolites with the highest fluctuation in response to the treatment of *L. minor* cultures with the *Bacillus* sp. PTA13 lipopeptide extract at concentration equal to the EC_25_ (156 μg·mL^−1^). Confidence intervals have been derived from jack-knifing (*p* < 95%). Figure S4: Variable influence on projection (VIP) plot displaying the VIP values of the annotated *Lemna minor* L. metabolites. High VIP values correspond to metabolites with the highest fluctuation in response to the treatment of *L. minor* cultures with the *Bacillus* sp. PTA13 lipopeptide extract at concentration equal to the EC_50_ (419 μg·mL^−1^). Confidence intervals have been derived from jack-knifing (*p* < 95%). Table S1: Composition of Steinberg medium for the cultivation of *Lemna minor* L. Table S2: Composition of the stock solutions for the preparation of Steinberg medium. Table S3: Metabolite features of *Lemna minor* L. GC/EI/MS total ion chromatograms (TIC) annotated in various identification levels.

## References

[1] Lykogianni M., Bempelou E., Karamaouna F., Aliferis K.A. (2021). Do pesticides promote or hinder sustainability in agriculture? The challenge of sustainable use of pesticides in modern agriculture. Sci. Total Environ..

[2] Carvalho F.P. (2006). Agriculture, pesticides, food security and food safety. Environ. Sci. Policy.

[3] Damalas C.A., Selamat J., Iqbal S.Z. (2016). Safe food production with minimum and judicious use of pesticides. Food Safety.

[4] Nicolopoulou-Stamati P., Maipas S., Kotampasi C., Stamatis P., Hens L. (2016). Chemical pesticides and human health: The urgent need for a new concept in agriculture. Front. Public Health.

[5] Aliferis K.A., Chrysayi-Tokousbalides M. (2011). Metabolomics in pesticide research and development: Review and future perspectives. Metabolomics.

[6] Mojiri A., Zhou J.L., Robinson B., Ohashi A., Ozaki N., Kindaichi T., Farraji H., Vakili M. (2020). Pesticides in aquatic environments and their removal by adsorption methods. Chemosphere.

[7] Gonzalez-Rey M., Tapie N., Le Menach K., Devier M.-H., Budzinski H., Bebianno M.J. (2015). Occurrence of pharmaceutical compounds and pesticides in aquatic systems. Mar. Pollut. Bull..

[8] deNoyelles F., Dewey S.L., Huggins D.G., Kettle W.D., Graney R.L. (2020). Aquatic mesocosms in ecological effects testing: Detecting direct and indirect effects of pesticides. Aquatic Mesocosm Studies in Ecological Risk Assessment.

[9] Fadiji A.E., Babalola O.O. (2020). Elucidating mechanisms of endophytes used in plant protection and other bioactivities with multifunctional prospects. Front. Bioeng. Biotechnol..

[10] Dini-Andreote F. (2020). Endophytes: The second layer of plant defense. Trends Plant Sci..

[11] Bamisile B.S., Dash C.K., Akutse K.S., Keppanan R., Wang L. (2018). Fungal endophytes: Beyond herbivore management. Front. Microbiol..

[12] Gouda S., Das G., Sen S.K., Shin H.-S., Patra J.K. (2016). Endophytes: A treasure house of bioactive compounds of medicinal importance. Front. Microbiol..

[13] De Silva N.I., Brooks S., Lumyong S., Hyde K.D. (2019). Use of endophytes as biocontrol agents. Fungal Biol. Rev..

[14] Khare E., Mishra J., Arora N.K. (2018). Multifaceted interactions between endophytes and plant: Developments and prospects. Front. Microbiol..

[15] Bastías D.A., Gianoli E., Gundel P.E. (2021). Fungal endophytes can eliminate the plant growth–defence trade-off. New Phytol..

[16] Rai N., Kumari Keshri P., Verma A., Kamble S.C., Mishra P., Barik S., Kumar Singh S., Gautam V. (2021). Plant associated fungal endophytes as a source of natural bioactive compounds. Mycology.

[17] Rustamova N., Bozorov K., Efferth T., Yili A. (2020). Novel secondary metabolites from endophytic fungi: Synthesis and biological properties. Phytochem. Rev..

[18] Gao H., Li G., Lou H.-X. (2018). Structural diversity and biological activities of novel secondary metabolites from endophytes. Molecules.

[19] Papadopoulou E.-A., Angelis A., Antoniadi L., Aliferis K.A., Skaltsounis A.-L. (2021). Discovering the Next-Generation Plant Protection Products: A Proof-of-Concept via the Isolation and Bioactivity Assessment of the Olive Tree Endophyte *Bacillus* sp. PTA13 Lipopeptides. Metabolites.

[20] Penha R.O., Vandenberghe L.P., Faulds C., Soccol V.T., Soccol C.R. (2020). *Bacillus* lipopeptides as powerful pest control agents for a more sustainable and healthy agriculture: Recent studies and innovations. Planta.

[21] Fazle Rabbee M., Baek K.-H. (2020). Antimicrobial activities of lipopeptides and polyketides of *Bacillus velezensis* for agricultural applications. Molecules.

[22] Olishevska S., Nickzad A., Déziel E. (2019). *Bacillus* and *Paenibacillus* secreted polyketides and peptides involved in controlling human and plant pathogens. Appl. Microbiol. Biotechnol..

[23] Sharma D., Singh S.S., Baindara P., Sharma S., Khatri N., Grover V., Patil P.B., Korpole S. (2020). Surfactin like broad spectrum antimicrobial lipopeptide co-produced with sublancin from *Bacillus subtilis* strain A52: Dual reservoir of bioactives. Front. Microbiol..

[24] Basaid K., Chebli B., Mayad E.H., Furze J.N., Bouharroud R., Krier F., Barakate M., Paulitz T. (2021). Biological activities of essential oils and lipopeptides applied to control plant pests and diseases: A review. Int. J. Pest Manag..

[25] Santos V.S.V., Silveira E., Pereira B.B. (2018). Toxicity and applications of surfactin for health and environmental biotechnology. J. Toxicol. Environ. Health Part B.

[26] Kostopoulou S., Ntatsi G., Arapis G., Aliferis K.A. (2020). Assessment of the effects of metribuzin, glyphosate, and their mixtures on the metabolism of the model plant *Lemna minor* L. applying metabolomics. Chemosphere.

[27] Aliferis K.A., Materzok S., Paziotou G.N., Chrysayi-Tokousbalides M. (2009). *Lemna minor* L. as a model organism for ecotoxicological studies performing ^1^H NMR fingerprinting. Chemosphere.

[28] OECD (2006). Test No. 221: Lemna sp. Growth Inhibition Test.

[29] ISO Water Quality—Determination of the Toxic Effect of Water Constituents and Waste Water on Duckweed (Lemna minor)—Duckweed Growth Inhibition Test. https://www.iso.org/standard/34074.html.

[30] Hiscox J., Israelstam G. (1979). A method for the extraction of chlorophyll from leaf tissue without maceration. Can. J. Bot..

[31] Arnon D.I. (1949). Copper enzymes in isolated chloroplasts. Polyphenoloxidase in *Beta vulgaris*. Plant Physiol..

[32] Kirk J., Allen R. (1965). Dependence of chloroplast pigment synthesis on protein synthesis: Effect of actidione. Biochem. Biophys. Res. Commun..

[33] Bekele E.A., Annaratone C.E., Hertog M.L., Nicolai B.M., Geeraerd A.H. (2014). Multi-response optimization of the extraction and derivatization protocol of selected polar metabolites from apple fruit tissue for GC–MS analysis. Anal. Chim. Acta.

[34] Fiehn O., Robertson D., Griffin J., van der Werf M., Nikolau B., Morrison N., Sumner L.W., Goodacre R., Hardy N.W., Taylor C. (2007). The metabolomics standards initiative (MSI). Metabolomics.

[35] Tsugawa H., Cajka T., Kind T., Ma Y., Higgins B., Ikeda K., Kanazawa M., VanderGheynst J., Fiehn O., Arita M. (2015). MS-DIAL: Data-independent MS/MS deconvolution for comprehensive metabolome analysis. Nat. Methods.

[36] Gitelson A. (2020). Towards a generic approach to remote non-invasive estimation of foliar carotenoid-to-chlorophyll ratio. J. Plant Physiol..

[37] Fekete-Kertész I., Kunglné-Nagy Z., Gruiz K., Magyar Á., Farkas É., Molnár M. (2015). Assessing toxicity of organic aquatic micropollutants based on the total chlorophyll content of *Lemna minor* as a sensitive endpoint. Period. Polytech. Chem. Eng..

[38] Hildebrandt T.M., Nesi A.N., Araújo W.L., Braun H.-P. (2015). Amino acid catabolism in plants. Mol. Plant.

[39] Wahman R., Cruzeiro C., Graßmann J., Schröder P., Letzel T. (2022). The changes in *Lemna minor* metabolomic profile: A response to diclofenac incubation. Chemosphere.

[40] Li R., Luo C., Qiu J., Li Y., Zhang H., Tan H. (2022). Metabolomic and transcriptomic investigation of the mechanism involved in enantioselective toxicity of imazamox in *Lemna minor*. J. Hazard. Mater..

[41] Rai V. (2002). Role of amino acids in plant responses to stresses. Biol. Plant..

[42] Rodrigues-Corrêa K.C.d.S., Fett-Neto A.G. (2019). Abiotic stresses and non-protein amino acids in plants. Crit. Rev. Plant Sci..

[43] Galili G. (2011). The aspartate-family pathway of plants: Linking production of essential amino acids with energy and stress regulation. Plant Signal. Behav..

[44] Okumoto S., Funck D., Trovato M., Forlani G. (2016). Amino acids of the glutamate family: Functions beyond primary metabolism. Front. Plant Sci..

[45] Brzezowski P., Richter A.S., Grimm B. (2015). Regulation and function of tetrapyrrole biosynthesis in plants and algae. Biochim. Biophys. Acta Bioenerg..

[46] Kirma M., Araújo W.L., Fernie A.R., Galili G. (2012). The multifaceted role of aspartate-family amino acids in plant metabolism. J. Exp. Bot..

[47] Han M., Zhang C., Suglo P., Sun S., Wang M., Su T. (2021). L-Aspartate: An essential metabolite for plant growth and stress acclimation. Molecules.

[48] Ros R., Muñoz-Bertomeu J., Krueger S. (2014). Serine in plants: Biosynthesis, metabolism, and functions. Trends Plant Sci..

[49] Ho C.-L., Saito K. (2001). Molecular biology of the plastidic phosphorylated serine biosynthetic pathway in *Arabidopsis thaliana*. Amino Acids.

[50] Cascales-Miñana B., Muñoz-Bertomeu J., Flores-Tornero M., Anoman A.D., Pertusa J., Alaiz M., Osorio S., Fernie A.R., Segura J., Ros R. (2013). The phosphorylated pathway of serine biosynthesis is essential both for male gametophyte and embryo development and for root growth in *Arabidopsis*. Plant Cell.

[51] Maeda H., Dudareva N. (2012). The shikimate pathway and aromatic amino acid biosynthesis in plants. Annu. Rev. Plant Biol..

[52] Bouche N., Fromm H. (2004). GABA in plants: Just a metabolite?. Trends Plant Sci..

[53] Zhang L., Sun C. (2018). Fengycins, cyclic lipopeptides from marine *Bacillus subtilis* strains, kill the plant-pathogenic fungus Magnaporthe grisea by inducing reactive oxygen species production and chromatin condensation. Appl. Environ. Microbiol..

[54] Wang Y., Zhang C., Liang J., Wu L., Gao W., Jiang J. (2020). Iturin A extracted from *Bacillus subtilis* WL-2 affects *Phytophthora*
*infestans* via cell structure disruption, oxidative stress, and energy supply dysfunction. Front. Microbiol..

[55] Weber H. (2002). Fatty acid-derived signals in plants. Trends Plant Sci..

[56] Aliferis K.A., Faubert D., Jabaji S. (2014). A metabolic profiling strategy for the dissection of plant defense against fungal pathogens. PLoS ONE.

[57] Wasternack C. (2014). Action of jasmonates in plant stress responses and development-applied aspects. Biotechnol. Adv..

[58] Ruan J., Zhou Y., Zhou M., Yan J., Khurshid M., Weng W., Cheng J., Zhang K. (2019). Jasmonic acid signaling pathway in plants. Int. J. Mol. Sci..

[59] Ghorbel M., Brini F., Sharma A., Landi M. (2021). Role of jasmonic acid in plants: The molecular point of view. Plant Cell Rep..

[60] Wang J., Song L., Gong X., Xu J., Li M. (2020). Functions of jasmonic acid in plant regulation and response to abiotic stress. Int. J. Mol. Sci..

[61] Henry G., Deleu M., Jourdan E., Thonart P., Ongena M. (2011). The bacterial lipopeptide surfactin targets the lipid fraction of the plant plasma membrane to trigger immune-related defence responses. Cell. Microbiol..

[62] Farace G., Fernandez O., Jacquens L., Coutte F., Krier F., Jacques P., Clément C., Barka E.A., Jacquard C., Dorey S. (2015). Cyclic lipopeptides from *Bacillus*
*subtilis* activate distinct patterns of defence responses in grapevine. Mol. Plant Pathol..

[63] Gond S.K., Bergen M.S., Torres M.S., White J.F. (2015). Endophytic *Bacillus* spp. produce antifungal lipopeptides and induce host defence gene expression in maize. Microbiol. Res..

[64] Pérez-García A., Romero D., De Vicente A. (2011). Plant protection and growth stimulation by microorganisms: Biotechnological applications of Bacilli in agriculture. Curr. Opin. Biotechnol..

[65] Falardeau J., Wise C., Novitsky L., Avis T.J. (2013). Ecological and mechanistic insights into the direct and indirect antimicrobial properties of *Bacillus subtilis* lipopeptides on plant pathogens. J. Chem. Ecol..

[66] Schulze J., Tesfaye M., Litjens R., Bucciarelli B., Trepp G., Miller S., Samac D., Allan D., Vance C. (2002). Malate plays a central role in plant nutrition. Plant Soil.

[67] Guo H., Chen H., Hong C., Jiang D., Zheng B. (2017). Exogenous malic acid alleviates cadmium toxicity in *Miscanthus sacchariflorus* through enhancing photosynthetic capacity and restraining ROS accumulation. Ecotoxicol. Environ. Saf..

[68] Araújo W.L., Nunes-Nesi A., Fernie A.R. (2011). Fumarate: Multiple functions of a simple metabolite. Phytochemistry.

[69] Chatterjee S., Kumar V. (2017). Quantitative systems pharmacology: Lessons from fumaric acid and herbal remedies. Drug Des..

[70] Ryan D.G., Murphy M.P., Frezza C., Prag H.A., Chouchani E.T., O’Neill L.A., Mills E.L. (2019). Coupling Krebs cycle metabolites to signalling in immunity and cancer. Nat. Metab..

[71] Erdemir U.S., Arslan H., Guleryuz G., Yaman M., Gucer S. (2018). Manganese tolerance in Verbascum olympicum Boiss. affecting elemental uptake and distribution: Changes in nicotinic acid levels under stress conditions. Environ. Sci. Pollut. Res..

[72] Ahmad Z., Bashir K., Matsui A., Tanaka M., Sasaki R., Oikawa A., Hirai M.Y., Zu Y., Kawai-Yamada M., Rashid B. (2021). Overexpression of nicotinamidase 3 (NIC3) gene and the exogenous application of nicotinic acid (NA) enhance drought tolerance and increase biomass in *Arabidopsis*. Plant Mol. Biol..

[73] Staykov N.S., Angelov M., Petrov V., Minkov P., Kanojia A., Guinan K.J., Alseekh S., Fernie A.R., Sujeeth N., Gechev T.S. (2020). An *Ascophyllum nodosum*-derived biostimulant protects model and crop plants from oxidative stress. Metabolites.

[74] Graf E. (1992). Antioxidant potential of ferulic acid. Free Radic. Biol. Med..

[75] Mandal S.M., Chakraborty D., Dey S. (2010). Phenolic acids act as signaling molecules in plant-microbe symbioses. Plant Signal. Behav..

[76] Lattanzio V., Lattanzio V.M., Cardinali A. (2006). Role of phenolics in the resistance mechanisms of plants against fungal pathogens and insects. Phytochem. Adv. Res..

[77] Goddijn O.J., van Dun K. (1999). Trehalose metabolism in plants. Trends Plant Sci..

[78] Elbein A.D., Pan Y., Pastuszak I., Carroll D. (2003). New insights on trehalose: A multifunctional molecule. Glycobiology.

[79] Eastmond P.J., Van Dijken A.J., Spielman M., Kerr A., Tissier A.F., Dickinson H.G., Jones J.D., Smeekens S.C., Graham I.A. (2002). Trehalose-6-phosphate synthase 1, which catalyses the first step in trehalose synthesis, is essential for *Arabidopsis* embryo maturation. Plant J..

[80] Satoh-Nagasawa N., Nagasawa N., Malcomber S., Sakai H., Jackson D. (2006). A trehalose metabolic enzyme controls inflorescence architecture in maize. Nature.

[81] Gómez L.D., Baud S., Gilday A., Li Y., Graham I.A. (2006). Delayed embryo development in the Arabidopsis trehalose-6-phosphate synthase 1 mutant is associated with altered cell wall structure, decreased cell division and starch accumulation. Plant J..

[82] John R., Raja V., Ahmad M., Jan N., Majeed U., Ahmad S., Yaqoob U., Kaul T., Sarwat M., Ahmad A., Abdin M.Z., Ibrahim M.M. (2016). Trehalose: Metabolism and role in stress signaling in plants. Stress Signaling in Plants: Genomics and Proteomics Perspective Volume 2.

[83] Banat I.M., Satpute S.K., Cameotra S.S., Patil R., Nyayanit N.V. (2014). Cost effective technologies and renewable substrates for biosurfactants’ production. Front. Microbiol..

[84] Chen W.C., Juang R.S., Wei Y.H. (2015). Applications of a lipopeptide biosurfactant, surfactin, produced by microorganisms. Biochem. Eng. J..

[85] Zhi Y., Wu Q., Xu Y. (2017). Genome and transcriptome analysis of surfactin biosynthesis in *Bacillus amyloliquefaciens* MT45. Sci. Rep..

[86] Paraszkiewicz K., Bernat P., Kuśmierska A., Chojniak J., Płaza G. (2018). Structural identification of lipopeptide biosurfactants produced by *Bacillus subtilis* strains grown on the media obtained from renewable natural resources. J. Environ. Manage..

[87] Jung J., Yu K.O., Ramzi A.B., Choe S.H., Kim S.W., Han S.O. (2012). Improvement of surfactin production in *Bacillus subtilis* using synthetic wastewater by overexpression of specific extracellular signaling peptides, *comX* and *phrC*. Biotechnol. Bioeng..

[88] Jiao S., Li X., Yu H., Yang H., Li X., Shen Z. (2017). In situ enhancement of surfactin biosynthesis in *Bacillus subtilis* using novel artificial inducible promoters. Biotechnol. Bioeng..

[89] Wang Q., Yu H., Wang M., Yang H., Shen Z. (2018). Enhanced biosynthesis and characterization of surfactin isoforms with engineered *Bacillus subtilis* through promoter replacement and *Vitreoscilla* hemoglobin co-expression. Process Biochem..

