# Supplementary material for: A Metabolomic Approach to Assess the Toxicity of the Olive Tree Endophyte Bacillus sp. PTA13 Lipopeptides to the Aquatic Macrophyte Lemna minor L
Source: Toxics. 2022 Aug 25;10(9):494. doi: 10.3390/toxics10090494 (PMC9505422; doi:10.3390/toxics10090494)
Supplement: Supplementary file 1 [file toxics-10-00494-s001.zip › Table S1.pdf]

Table S1. Composition of Steinberg medium for the cultivation of *Lemna minor* L.

**Macroelements**	**M.W.**	**mg L^{-1}**	**mmol L^{-1}**
KNO_3	101.12	350.00	3.46
$Ca(NO_3)_2$ □ $4H_2O$	236.15	295.00	1.25
KH_2PO_4	136.09	90.00	0.66
K_2HPO_4	174.18	12.60	0.072
$MgSO_4$ □ $7H_2O$	246.37	100.00	0.41
**Microelements**	**M.W.**	**mg L^{-1}**	**mmol L^{-1}**
H_3BO_3	61.83	120.00	1.94
$ZnSO_4$ □ $7H_2O$	287.43	180.00	0.63
Na_2MoO_4 □ $2H_2O$	241.92	44.00	0.18
$MnCl_2$ □ $4H_2O$	197.84	180.00	0.91
$FeCl_3$ □ $6H_2O$	270.21	760.00	2.81
EDTA Disodium-dihydrate	372.24	1500.00	4.03
