# Supplementary material for: A Metabolomic Approach to Assess the Toxicity of the Olive Tree Endophyte Bacillus sp. PTA13 Lipopeptides to the Aquatic Macrophyte Lemna minor L
Source: Toxics. 2022 Aug 25;10(9):494. doi: 10.3390/toxics10090494 (PMC9505422; doi:10.3390/toxics10090494)
Supplement: Supplementary file 1 [file toxics-10-00494-s001.zip › Table S2.pdf]

Table S2. Composition of the stock solutions for the preparation of Steinberg medium.

Stock Solution	Macroelements	g L^{-1}
I	KNO_3	17,50
	KH_2PO_4	4,50
	K_2HPO_4	0,63
II	$MgSO_4$ □ $7H_2O$	5,00
III	$Ca(NO_3)_2$ □ $4H_2O$	14,75
Stock Solution	Microelements	mg L^{-1}
IV	H_3BO_3	120,00
V	$ZnSO_4$ □ $7H_2O$	180,00
VI	Na_2MoO_4 □ $2H_2O$	44,00
VII	$MnCl_2$ □ $4H_2O$	180
VIII	$FeCl_3$ □ $6H_2O$	760,00
	EDTA Disodium-dihydrate	1.500,00
