# Supplementary figures and images for: A Metabolomic Approach to Assess the Toxicity of the Olive Tree Endophyte Bacillus sp. PTA13 Lipopeptides to the Aquatic Macrophyte Lemna minor L
Source: Toxics. 2022 Aug 25;10(9):494. doi: 10.3390/toxics10090494 (PMC9505422; doi:10.3390/toxics10090494)

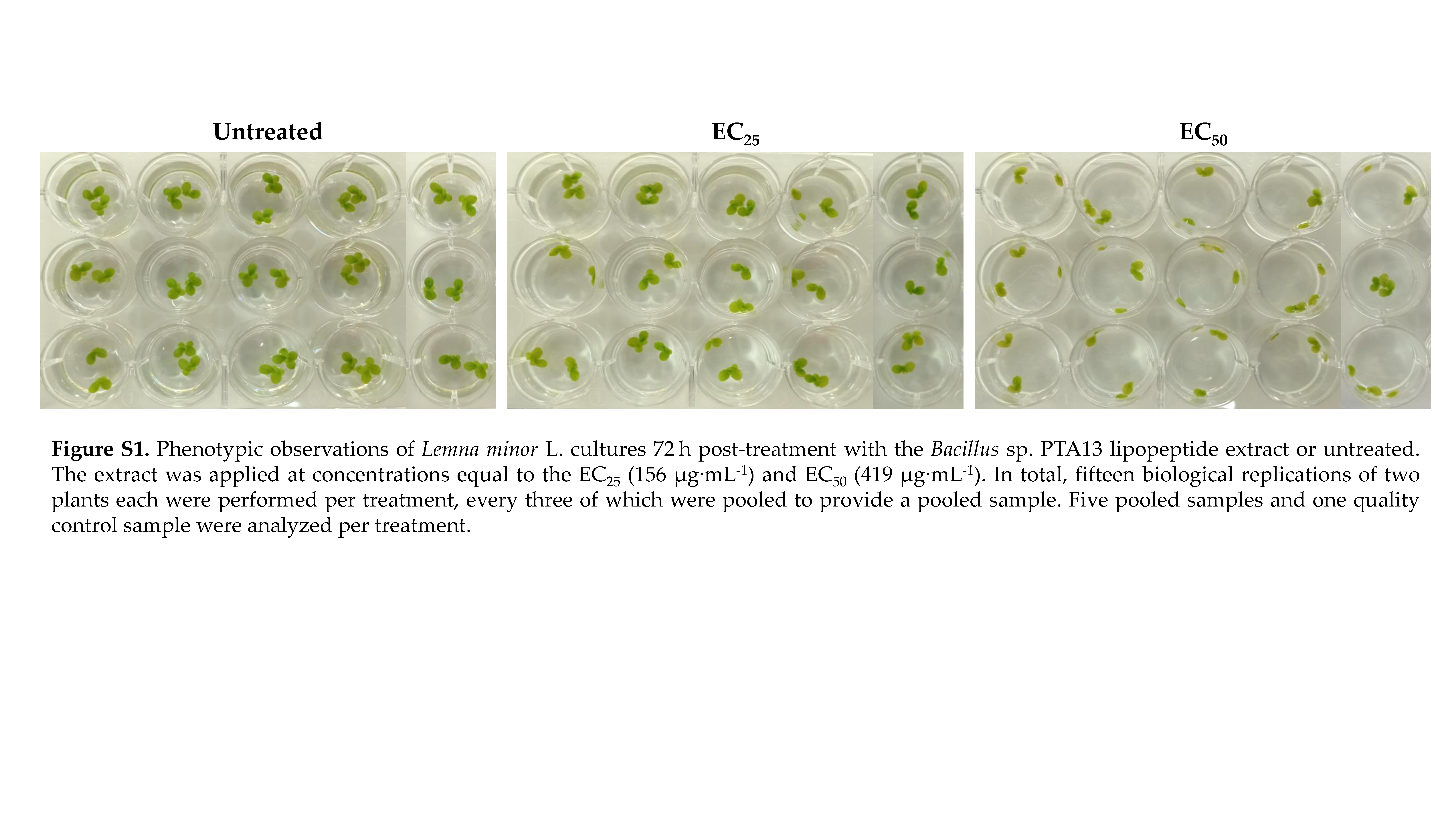

Supplement: Supplementary file 1 [file toxics-10-00494-s001.zip › Figure S1.jpg]

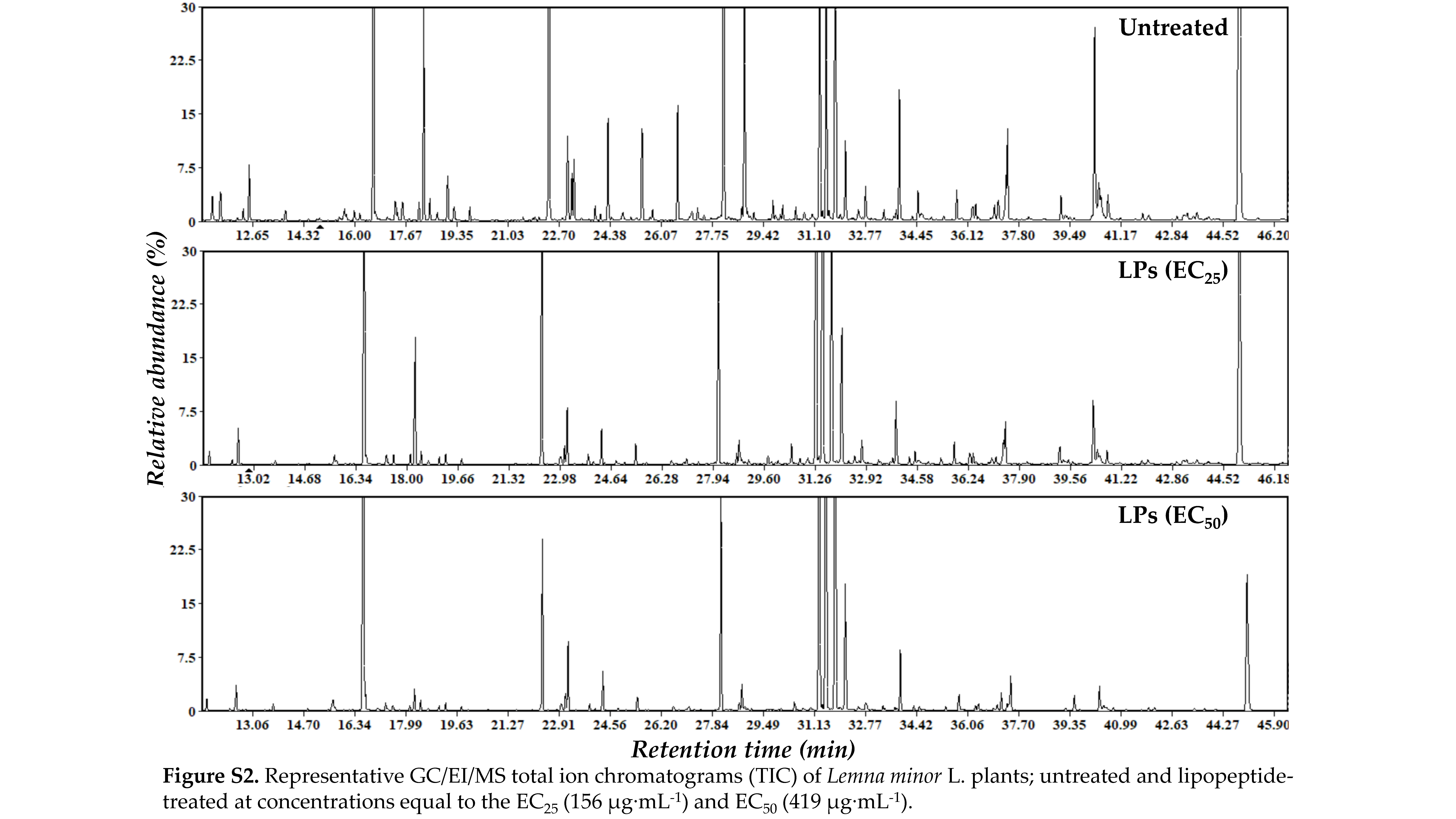

Supplement: Supplementary file 1 [file toxics-10-00494-s001.zip › Figure S2.jpg]

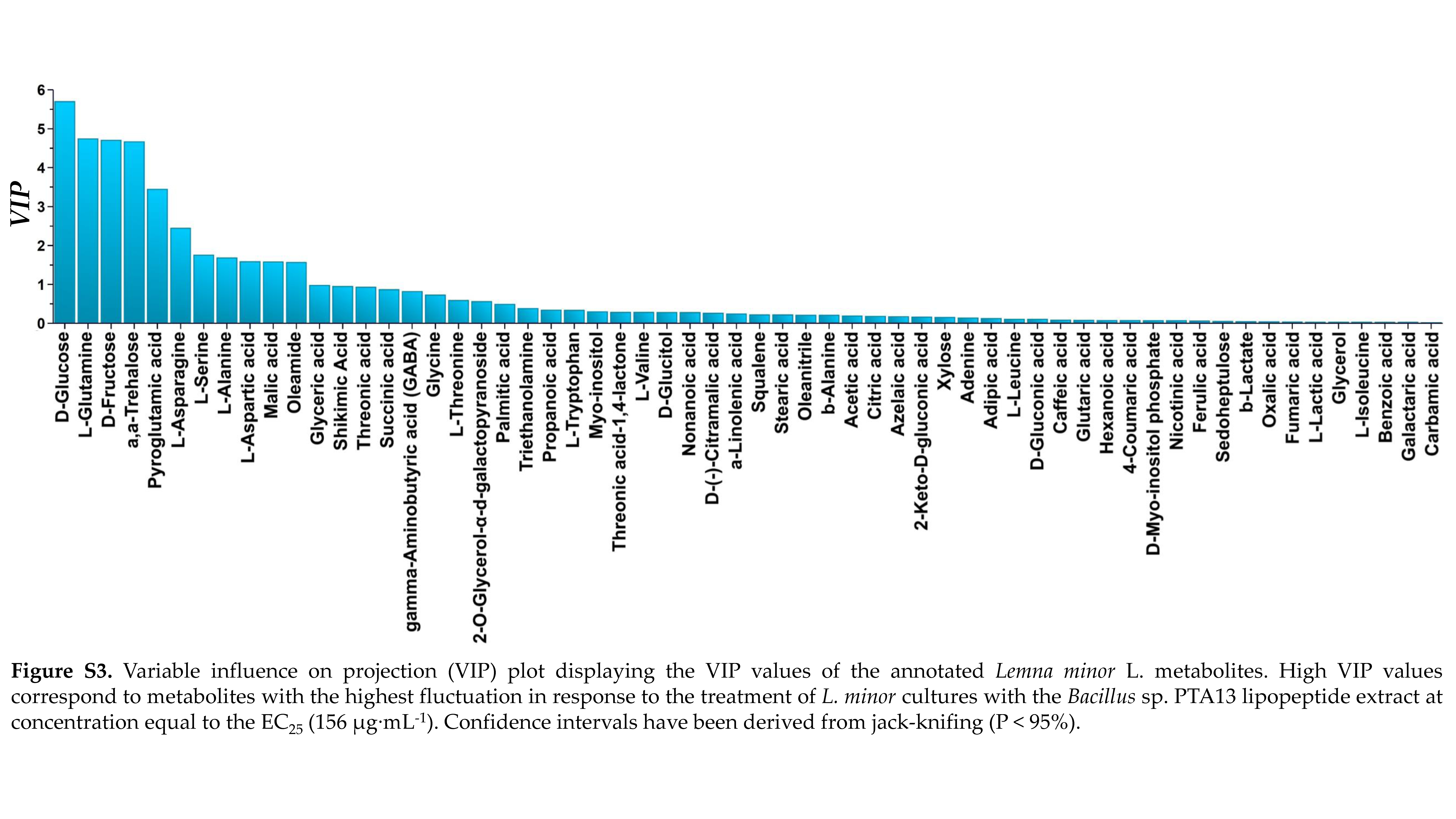

Supplement: Supplementary file 1 [file toxics-10-00494-s001.zip › Figure S3.jpg]

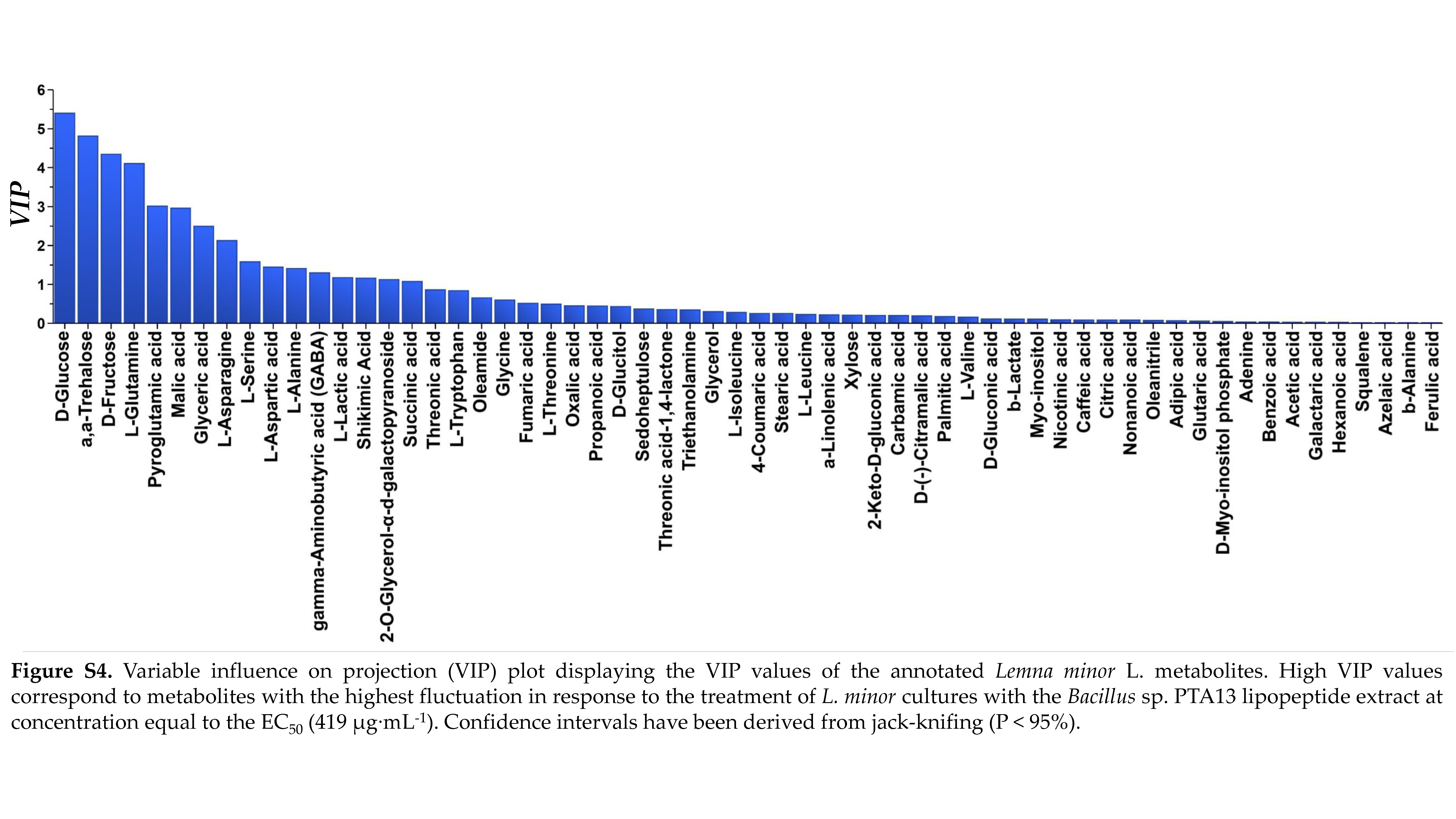

Supplement: Supplementary file 1 [file toxics-10-00494-s001.zip › Figure S4.jpg]
